# Contemporary Strategies in the Management of Civilian Neck Zone II Vascular Trauma

**DOI:** 10.3389/fsurg.2017.00056

**Published:** 2017-09-29

**Authors:** Georgios Karaolanis, Konstantinos Maltezos, Chris Bakoyiannis, Sotiris Georgopoulos

**Affiliations:** ^1^First Department of Surgery, Division of Vascular Surgery, National and Kapodistrian University of Athens, Athens, Greece

**Keywords:** cervical vascular trauma, arterial injuries, venous injuries, open repair, endovascular repair, conservative treatment

## Abstract

Neck trauma is the leading cause of death mainly in younger persons posing to surgeons the dilemma whether to proceed with reconstruction of vascular injuries either in the presence of coma or in severe neurological deficit. Vascular injuries in zone II predominate over the other injuries located in zones I/III of the neck. Conventional open repair of carotid injuries with primary closure or interposition grafting is always recommended due to the effective long-term results for penetrating injuries or for patients unfit for endovascular intervention. In cases of blunt trauma, anticoagulation or antiplatelet therapy should be administered first in neurologically stable patients. In case of worsening of the neurological status of the patient despite adequate anticoagulation endovascular means should be considered in cases of appropriate anatomy of the arterial trauma. We provide an update on penetrating/blunt trauma in zone II of the neck, giving emphasis on the anticoagulant and endovascular treatment.

## Introduction

Neck trauma is the leading cause of death in younger persons, posing to surgeons the dilemma whether to proceed with reconstruction of vascular injuries either in the presence of coma or in severe neurological deficit (1). Most patients presenting with vascular neck injuries passed away during injury or immediately after transportation to the medical center (2). For better evaluation and approach of the injury, the neck was separated into three anatomical zones: zone I (sternal notch to cricoid cartilage), zone II (cricoid cartilage to angle of the mandible), and zone III angle (mandible to the base of skull) (3). Usually, the majority of injuries are found in zone II (47%) whereas a smaller amount of injuries can be found in zone III (19%) and zone I (18%). Vascular vessels such as the carotid artery (CA) situated in this zone are commonly injured, and it is associated with a high mortality rate (50%) and incidence of neurological deficit development (80%) (4).

Newer diagnostic and therapeutic modalities have simplified care and the need for diagnostic neck exploration. Currently, high-quality computed tomography angiography (CTA) is a quick and accurate method identifying the vast majority of injuries prior to any operative intervention (5). Experienced vascular trauma centers described their experience; however, their point of view concerning CTA is still unclear (2). This review aims to examine treatment of vascular injuries limited in zone II of the neck and we hope that the juxtaposed discussions will shed light on this controversial topic; hopefully, some global insights will emerge.

### Penetrating Injuries (PIs) in Zone II

Anatomical boundaries of zone II are the thoracic outlet and the angle of mandible. Commonly, the main vascular structures in this zone are the common carotid artery (CCA), the internal carotid artery (ICA), the external carotid artery (ECA), the internal jugular vein (IJV), and the vertebral artery (VA) (6). The patient presents often in the emergency room (ER) with “hard signs” (Table 1) that obviously require immediate exploration or “soft signs” that need further diagnostic evaluation and observation of the patient’s hemodynamic status. Hard signs are defined as shock, pulsatile bleeding mass, souffle, expanding hematoma, and loss of pulse with stable or developing neurological deficit. Soft signs include stable hematoma and history of bleeding at the scene of injury (6).

**Table 1:** Hard signs for immediate surgery after penetrating neck trauma.

Shock Pulsatile bleeding Expanding hematoma Audible bruit or palpable thrill Airway compromise Wound bubbling Extensive subcutaneous emphysema Stridor Hoarseness Signs of stroke or cerebral ischemia

Penetrating injuries are associated with high mortality due to the arterial damage. A total of 80% of deaths is the result of ischemic stroke while the remainder the result of exsanguination (7). A penetrating wound in the main vessels (CCA and IJV) may lead to loss of the airway and early asphyxiation due to the presence of large hematoma, which externally compresses or due to the presence of active hemorrhage originating from those vessels simultaneously with injury to the trachea. Most injuries are caused from firearms (45% of PIs), while the incidence of stab wounds and shotgun injuries is 40 and 4%, respectively (7).

The management of zone II injuries has been an object of debate over the previous decades (8). Conventionally, injuries in this zone would mandate immediate surgical exploration due to the accessible anatomy and high mortality of missed injuries (9). Bishara et al. (9) pointed out the possibility of missed injury in case an exploration is not carried out. However, mandatory exploration has been questioned compared to selective or non-operative management with serial examination and further diagnostic testing in asymptomatic, hemodynamically stable patients (10). Several studies from experienced trauma centers confirmed that zone II injuries can be reliably estimated during physical examination, thus leading to the confirmation or exclusion of vascular injuries (11, 12). The missed injury percentage is estimated at 0.7, questioning even the need for diagnostic imaging, such as angiography.

#### Diagnostic Evaluation

The treatment of penetrating neck injuries constitutes an important diagnostic and therapeutic challenge for trauma surgeons. Arteriogram is the golden rule of the detection of arterial pathology. Meyer and colleagues (13) compared the use of arteriogram with the conventional exploration. As a result, the authors concluded that arteriography was highly accurate, whereas the negative exploration rate was unacceptably high (69%). These results obviously reinforced the need for a more selective approach, presumably based on angiographic findings. However, its routine use has also been questioned because of the low rate of positive examination and the risk of complications in the access site (6). Despite these potential disadvantages of the method, we should acknowledge its therapeutic contribution, allowing for embolization of smaller vessels or stent grafting of larger arteries.

Nowadays, there is a trend toward less invasive techniques to diagnose vascular injuries. Lately, spiral and multidetector row computed tomography angiography (CTA) has proved to be a reliable technique for the evaluation of penetrating neck injuries. Múnera et al. (14) reported that CTA has a sensitivity of 90%, a specificity of 100%, a positive predictive value of 100%, and a negative predictive value of 98% compared to conventional angiography. Therefore, CTA along with physical examination is recommended as part of the examination of patients with “soft signs” as well as in cases of permanent hard signs that could delay a direct/urgent operation (15).

Another useful diagnostic modality in the evaluation of cervical trauma is Color Doppler Ultrasonography (CDU). This method could be used in stable patients with “soft signs” providing anatomical and hemodynamic information of the injured vessel. Numerous studies documented the ease and accuracy of the technique when applied to patients with penetrating wounds in zone II (6, 16, 17). However, CDU remains operator dependent and restricted only in zone II.

#### Surgical Treatment

The hemodynamic status of the patient on admission is the key point regarding management strategy. It is generally accepted that patients’ stability permits physical examination and/or CTA while unstable patients with expanding hematoma, active bleeding, or hypotension unresponsive to initial resuscitation should be driven immediately to the operating room. Surgical treatment after PI is still the main choice. Once an injury has been detected and controlled, the surgeon is called to decide whether to ligate, repair, or temporary shunt the vessel. The vessel most commonly injured by penetrating mechanism is the IJV followed by CCA. The IJV and the ECA may be ligated with limited morbidity. On the contrary, ligation is not an acceptable surgical approach for ICA (18). Before 1950, ligation was a common technique for the management of carotid injuries, leading to a 30% stroke rate (18). Subsequent civilian series have documented reasonable short- and mid-term results (19). However, if at all possible, ligation of the CCA and ICA should be avoided due to the high rate of procedural mortality (45%).

The method of reconstruction of the injured cervical vessel depends on various factors such as the nature and extension of the injury, degree of contamination, and the surgeon’s preference. The great saphenous vein matches the size of the ICA and has excellent patency rate and limited infectious risk when it is used (20). Other surgical approaches are the transposition of the ECA to the ICA for injuries in the proximal part of it or the use of polytetrafluoroethylene (PTFE) and Dacron that both have equal patency (20). If gross contamination is present, CCA reconstruction could be performed using the superficial femoral artery (SFA) harvested from the groin. Interposition of PTFE graft in the harvested SFA is required (20). Shunting is recommended for ICA repair to minimize ongoing cerebral ischemia, although clamping without shunt could be used in CCA repair due to collateral flow from the ECA. After repair of the vascular injury, all patients must be closely monitored for signs of cerebral edema and intracranial hypertension (17).

Penetrating injuries to the vertebral arteries (VAs) are rare, with an incidence of less than 1% of all vascular injuries (21). These injuries tend to be ignored in the initial evaluation unless any major complication such as exsanguination or airway compromise is observed. VA is divided into four parts (V1–V4). V2 and V3 are the main segments included in zone II. All segments are difficult to access surgically and for the most part open exposure is contraindicated (21). In a recent study of traumatic VA injury due to PIs, 20 out of 92 patients required emergency surgery because of instability; ligation was used to achieve hemostasis (21). The rest was treated conservatively or with endovascular means.

### Blunt Injuries in Zone II

Blunt arterial injury in zone II is usually not clearly evident compared to penetrating trauma, thus its diagnosis may be delayed. In 1872, the first article concerning blunt carotid artery injury (BCAI) was circulated by Verniuel (22). Since then, knowledge on pathophysiology, screening, diagnosis, and treatment has been accumulated mainly over the past 30 years (23, 24).

The most commonly injured vessel is the extracranial part of the ICA, presenting with a low incidence rate (0.24–0.35%); however, increasing rates of morbidity (16–58%) and mortality (15–31%) are observed. In the majority of cases (76%), the leading cause of mortality is stroke (14, 25).

#### Trauma Mechanisms

The proposed mechanisms of ICA include (1) extreme hyperflexion/hyperextension and rotation of the neck leading to vessel stretch injury (2) vessel laceration from bony fracture (3) a direct blow to the vessel (26). Once the neck undergoes extreme movement, the contralateral carotid is put at risk, as it can be stretched against the cervical vertebral bodies. Unfortunately, injuries in the ICA are involved at or above the base of skull in 94% of cases (14). The ICA may also be injured by styloid process during sudden rotation or compressed by angle of mandible during hyperflexion (zone III). In this zone, the only option for treatment may be endovascular exclusion due to the high rate of morbidity and mortality in open surgery (27). A direct blow to the ICA in zone II can typically occur when the seat belt is not placed properly in case of a car accident or in case of hanging (14, 25).

It is estimated that BCAI are related to several vascular injuries such as intimal flap/dissection, occlusion/thrombosis, pseudoaneurysm complete transection, carotid cavernous fistula (zone III), or a combination of these lesions (28). Most authors agree that the most common mechanism of blunt trauma is extreme hyperflexion/hyperextension and rotation of the neck leading to ICA stretch injury (14). In this situation, lateral masses of the third and fourth cervical vertebrae produce an intimal tear, along the contralateral vessel. As a result, a false lumen appears from the circulating blood, which penetrates into the arterial wall through this damage and subsequently creates an intramural clot. Gradually, the true lumen is being compressed by the false lumen, whereas the intramural clot extends subadventitially leading to dissecting aneurysm or embolizes the intracranial vessels.

#### Symptoms and Signs

It is well known that BCAI have an increased percentage of devastating neurological morbidity (60%) and mortality (19–43%) (28–30). It is possible that clinical symptoms and signs do not appear immediately but hours and weeks following the injury (31, 32). More often, BCAI symptoms include transient ischemic attack (TIA) or stroke at the time of presentation. Other symptoms that can be observed are ipsilateral headache (58–92%), Horner’s syndrome (9–75%), neck pain (18–46%), bruits (12–39%), and tinnitus (13%) (33). Some experts reported that patients with localized symptoms (neck pain, Horner’s syndrome, and tinnitus) develop TIA and stroke (30 and 43%, respectively). Patients with severe neurological manifestations (TIA) on admission develop stroke 6 h to 31 days after injury. The majority of the ischemic stroke events are of embolic origin (34).

#### Diagnostic Evaluation

The cases of BCAI are not common but require immediate diagnosis; therefore, the identification of patients with blunt carotid injury is a challenging task. Berne et al. (35) highlighted the need for early assessment and diagnosis of patients suffering from carotid blunt trauma. They reported that patients with delayed diagnosis had a mortality rate of 80% (35). Thus, the need for screening criteria to prevent complications related to BCAI emerged.

First, the Denver and Memphis group proposed screening criteria that have gained subsequently wide acceptance (14, 36). These criteria were based on signs and/or symptoms and the presence of risk factors (Table 2). Later, the same scientific group proposed additional predictive factors such as mandible fractures, frontal skull fractures, diffuse axonal injury with Glasgow Coma Scale < 6 and thoracic injuries, and cardiac or great vessel injuries (33, 37). Another proposed scoring system was the Injury Severity Score (ISS). Recently, a study that used ISS reported that 2.7% of the patients with ISS score ≥16 were identified as having BCAI (38).

**Table 2:** Screening criteria for blunt carotid artery injury proposed by Denver and Memphis group.

Signs and symptoms	Risk factors
Arterial hemorrhage (neck/nose/mouth) Cervical bruit (patient <50 years) Expanding bleeding mass Focal neurological deficits (TIA, hemiparesis, vertebrobasilar symptoms, and Horner’s syndrome) Neurological signs not explained by brain imaging Stroke featured by CT/MRI	Le Fort II/III fractures Skull base fractures Mandible fractures Frontal skull fractures Occipital condyle fractures Carotid canal fractures Cervical spine injuries Anoxic brain injuries for hanging Seat belt sign Diffuse axonal injury with GCS < 6 Thoracic/cardiac and great vessels injury

*GCS, Glasgow Coma Scale; TIA, transient ischemic attack*.

Thus, with the use of the abovementioned screening criteria and risk factors, a significant improvement in detecting CA injury was observed. Miller et al. (37) using their proposed criteria in 216 patients with BCAI had a 29% diagnostic yield of blunt carotid and/or VA injuries. It is worth mentioned that many studies mentioned the cost of using these screening criteria. They concluded that the abovementioned criteria are cost-effective and with improved neurological outcome and survival (39).

For the investigation of BCAI, four imaging modalities have been proposed in the literature: digital subtraction arteriography (DSA), computed tomography angiography (CTA), magnetic resonance angiography (MRA), and Doppler ultrasonography. DSA remains the gold standard for diagnosis. However, due to its invasiveness, it is associated with low risk of stroke, and it is wide used to depict the extent and the severity of vessel injury. Assuming that various injuries grades may exhibit different complications with regard to response to therapy and ultimate neurological outcome, Biffl and colleagues (40) have proposed an arteriographic scale for BCAI (Table 3). Despite heparin administration, 5% of grade I injury progressed to grade III; 66% of grade II progressed to grade III or IV. The use of heparin treated just 4% of grade III injuries, whereas in 81% of the cases a surgery is needed. In the same study, patients with grade IV injuries did not resolve with heparinization, whereas 63% of grade V injury patients died. Later, Edwards and associates (41) showed an increased rate of healing for grade I/II lesions through the comparison of diagnostic arteriograms with follow-up arteriograms.

**Table 3:** Denver “grading scale for blunt carotid injury” adapted from Biffl and associates.

Grade I	Arteriographic appearance of irregularity of the vessel wall or a dissection/intramural hematoma with less than 25% luminal stenosis
Grade II	Intraluminal thrombus or raised intimal flap is visualized, or dissection/intramural hematoma with 25% or more luminal narrowing
Grade III	Pseudoaneurysm
Grade IV	Vessel occlusion
Grade V	Transection with free extravasation

Computed tomography angiography (CTA) is by far the most commonly preferred screening imaging method (60%) followed by MRI/MRA (22%), DSA (15%), and CDU (1.7%) (89). This results from various factors such as the more rapid availability. However, several authors believe that CTA is not as reliable and accurate as DSA, it has significant false-negative rate and underestimates the severity of injury (90). To circumvent this issue, recent studies have performed CTA and DSA in all patients being screened. Malhotra et al. (90) compared CTA and DSA in 92 patients undergoing screening for BCAI. Using DSA as diagnostic tool, they revealed four cases with BCAI, which had normal CTA. The sensitivity and specificity of CTA was 67 and 96%, respectively, with positive and negative predictive values reaching 73 and 95%, respectively. Similarly, the same conclusions were reported by the Memphis group, which examined 684 patients who underwent both CTA and DSA. They discovered 128 missed diagnoses by CTA (74). The authors concluded that CTA displays an unsatisfactory sensitivity (51%) in detecting BCAI, and it is proposed as an unreliable screening tool (91). Furthermore, some known limitations of CTA such as the inherent radiation exposure, need for patient transfer of the ER to radiology department, and the use of metal or dental artifact that alter the image quality must be highlighted.

Magnetic resonance angiography is proposed as a non-invasive imaging technique that provides data regarding the vessel structure and blood flow. Its strength point is the early identification of a cerebral ischemic infraction (92). MRA is not widely performed in clinical practice, in patients with signs of BCAI because it is an expensive technique or it is not available in the majority of medical centers (92).

Color duplex ultrasound is insufficient as an initial imaging technique, because of its low sensitivity (38%). Nevertheless, it is considered a helpful diagnostic tool in the follow-up of these patients (92).

#### Treatment

There have been no randomized controlled trials to establish optimum management of BCAI. The available treatment modalities are anticoagulation/antiplatelet therapy, open surgery, and endovascular treatment, which has gained support over the past years. Main aim of the treatment is to minimize the progression of the vessel injury, to decrease the incidence of ischemic events in asymptomatic patients, and to improve the overall neurological and survival outcomes.

A growing consensus concerning the use of antiplatelet and/or anticoagulants exists in the literature for lesions I or II (41). The major risk of patients suffering from these lesions is the development of stroke. Five percent of grade I injury evolved to grade III; 66% of grade II progressed to grade III or IV (41). About 63% of the patients injuries (lesions I–II) were resolved with observation compared to 70% of patients treated with anticoagulants. Both Western and Eastern Associations of Trauma Surgery recommended antithrombotic therapy for these grades of lesions unless any contraindication to anticoagulants exist (93). For grade II lesions, it has been noted that in 11% of cases under observation, a stroke was observed whereas resolution has been noted in 10% treated with anticoagulants (40). On follow-up for these lesions, with DSA used as diagnostic tool, the use of anticoagulants led to the treatment of 57% of grade I and 8% of grade II patients, whereas 8 and 43% for lesions I and II, respectively, progressed to lesion III (94). Due to the capability of these injuries to progress, some experts proposed follow-up using angiography in 7–10 days in the case of new onset of neurological symptoms (93).

Until today, there is no evidence regarding the superiority of both heparin and antiplatelet therapy to minimize the risk of stroke in asymptomatic patients or to improve the neurological status in those with symptoms. Several retrospective studies revealed that patients who were only observed developed more ischemic events compared with those who received anticoagulant treatment (41, 95). However, anticoagulant treatment carries the risk of intracranial hemorrhage (ICH) in few studies (41, 95). The rate of new developed hematoma in patients with cervical artery dissection without preexisting ICH is accounted to 3.5% in a recent study (46). The authors of this study concluded that antithrombotic therapy is relatively safe even in the presence of ICH. Some authors have advocated the antiplatelet’s use due to the lower bleeding rate compared to anticoagulants and they suggested that patients with persistent injuries after close imaging follow-up are considered for life-long aspirin administration (96).

Nowadays, open surgical approach for BCAI is rare; however, several techniques are available. Decision factors for the surgeon to proceed in open repair are the formation of thrombus of the injured CA, the state of the collateral circulation to the brain, presentation of expanding hematoma or aggravating neurological symptoms despite anticoagulants administration (96, 97). Carotid ligation in association with the deposition of permanent balloon occlusion in the proximal ICA has been proposed. However, the risk of delayed ischemia due to the propagation of thrombus or embolization events remains a dreadful consequence. Carotid ligation is considered an acceptable alternative in some patients; however, they run a high risk of cerebral ischemia and probably intracranial aneurysm development in the following years (97). In cases of bilateral BCAI, the sacrifice of the ICA would obviously be contraindicated.

Internal carotid artery pseudoaneurysms may develop and lead to symptoms despite conservative treatment. Although open surgical attempts to exclude an ICA pseudoaneurysm have been reported, during the past years endovascular technique remains the main way to face such kind of complications.

The birth of the endovascular technique in BCAI was in the mid-1990s, following its use in the coronary arteries field (48). The use of stents, coils, or even stents grafts can protect both against vessel-lumen and vessel-wall complications such as dissection, pseudoaneurysm, thrombosis, or even hemorrhage. The two main types of self-expandable stents (closed cells/open cells) promote laminal flow through the carotid lumen and they are completely covered with endothelium 3–6 weeks after placement. Except of the epithelialization, another benefit of the endovascular technique is the use of uncovered stents in association with the use of catheter-directed coil to exclude a pseudoaneurysm. The stent acts as a barrier, confirming the coils to pseudoaneurysm, securing its sufficient embolism, and prohibiting the outflow of these materials into the vital CA.

Endovascular treatment is being ideally accepted in cases of grade III or more serious injuries that cannot be medically treated. The reported resolution rate for grade III/IV with antithrombotic therapy alone is low which makes endovascular approach necessary. The main indications for endovascular treatment are (1) failed medical therapy (new ischemic attack, progression of the initial neurological symptomatology, or a continuing enlargement of carotid aneurysm) (2), new stroke event (3), and contraindication to medical treatment (98).

Biffl et al. (99) recommended stent placement in grade II or III that they have an increased risk of progressive stenosis or vessel occlusion. According to a literature review by DuBose (100) on the use of stents in carotid injuries treatment, the follow-up patency reached 79% while the rate of neurological disorders that followed the use of stent reached 3.5% (100). Under these circumstances, the initial benefits of stent placement are clearly described (101).

Until today, sporadic cases of endovascular repair (41, 42, 47, 49, 50, 52–62, 64, 65, 67–69, 71–78, 80, 81, 84, 85, 98, 102, 103) have been reported (Table 3). According to an encouraging report by the Denver group (72) concerning endovascular therapy for the treatment of type III lesions, 14 patients showed positive outcomes. However, later the same group reported a 45% carotid occlusion in 23 patients who had undergone stenting (54). In this study, patients with BCAI and pseudoaneurysm during an 8.5-year period were included. Twenty-three patients were treated with antithrombotic therapy and 23 with stents. Eight patients in the stent group and one patient in the antithrombotic group developed CA occlusion on follow-up angiography. The authors concluded that carotid stenting cannot be considered as a safe alternate to antithrombotic alone therapy.

Nevertheless, this study included several limitations. To begin with, the treatment took place from 1996 to 2001 when the shortage of dual-antiplatelet therapy could lead to an increased percentage of in-stent thrombosis. The second limitation concerns treatment selection criteria. The head surgeon was responsible for the selection of stenting over conservative therapy. This could be considered as selection bias for stenting in more complex situations (54). Moreover, both Duane et al. (65) and Parodi et al. (76) described a case of stent graft occlusion. Both occlusions occurred due to the cessation of anticoagulants treatment.

Berne et al. (48) assessed early anticoagulation alone compared to stent placement in combination with antiplatelets grade III carotid injuries and pseudoaneurysms. A total of 11 patients participated in the study and were treated with stents. All the patients received dual-antiplatelet regimens (clopidogrel 75 mg, and aspirin 80 mg, daily). Two deaths were noted in the study because of cerebral edema; acute closed head injuries were also observed in these patients. All nine patients who survived did not present with thrombosis during a 4-year follow-up period. The authors deduced that timely anticoagulation treatment with CA stenting is a secure and efficient option for the treatment of non-occlusive injuries and pseudoaneurysms.

Subsequently, Edwards et al. (41) published their experience in 18 patients who suffered from BCAI. Fourteen of them were available for a mean follow-up of 29.7 months and were free of stent thrombosis. In another cases series, Cohen et al. reported their experience with endovascular treatment of 10 patients with traumatic dissection of the CA. No peri-procedural complications were observed. At sonographic follow-up up to 28 months, no in-stent thrombosis was noted. The authors proposed the endovascular stenting as a rational and effective way to restore the artery lumen in selected patients.

Recently, Seth et al. (42) retrospectively published their results in 47 blunt/penetrating traumatic carotid injury patients who were treated with endovascular means. Twenty-one of them initially underwent endovascular repair following medical treatment. In that population, the rate of non-flow limiting grade III lesions stood at 87.5% while non-flow limiting grade II lesions stood at 14.3%. Vessel lumen was successfully restored in 50% while in the remaining 50% it was evaluated as satisfactory to good. Only one case with complete stent occlusion was observed but the patient remained asymptomatic.

A recent meta-analysis (98) was conducted with the aim to evaluate the safety and efficacy of endovascular intervention for BCAI. The researchers found 153 extra cranial CA injuries in 140 cases of treatment with endovascular techniques. The reported technical success was high (99%) in this series. In 60 vessels (98.4%) of pseudoaneurysm cases out of 61, the stent placement was successful or occlusion was performed when suited. Only 2 patients out of 140 developed neurological sequelae during a mean follow-up of 17.7 months. The rest of patients demonstrated either unchanged or improved neurological status.

Grade IV and V lesions are challenging for the endovascular technique and may be accompanied by open surgical intervention. Grade IV lesions automatically entail total vessel occlusion. The passage of endovascular catheter through this type of lesions may be technically complicated. In one case, the authors reported their experience in such kind of lesions (grade IV) using coaxial microcatheters and soft-tip micro-guide; however, this is the only case described in the literature (103).

Grade V lesions with vessel transection and frank extravasation are highly lethal, with the mortality rate approaching 100% and require immediate surgical intervention whenever possible. Due to the rarity of clinical data and the absence of prospective randomized studies comparing endovascular to open surgical approach for lesions V, no safe conclusions could be extracted for the superiority of each technique. However, grade V BCAI require immediate, sufficient surgical intervention similar to that required for penetrating carotid injuries.

To sum up, endovascular approach seems to be feasible in type III lesions in combination with antiplatelet agents, while type IV lesions have poor outcomes and sparse attempts are still secluded. Table 4 summarizes the main studies and their results.

**Table 4:** The main published studies after penetrating/blunt injuries in zone II of the neck.

Reference	Year	Type of study	Number of patients included in the study	Number of patients injured in zone II (%)	Injured vessel *n* %	Cause of trauma	Conservative treatment	Surgical therapy	Endovascular therapy	Overall mortality in study (%)
Seth et al. (42)	2013	RS	47	100	ICA	BI/PI	+		+	0
Kim et al. (43)	2011	CS	2	100	CCA/ICA	PI			+	0
Reva et al. (44)	2010	RS	17	89	CCA/ICA/IJV/EJV	PI		+		28
Testerman et al. (45)	2010	CR	1	100	ICA	BI			+	0
Hinson et al. (46)	2010	RS	55	NR	ICA/CCA	BI	+			0
Chaer et al. (47)	2008	CR	1	100	ICA	BI	+		+	0
Berne et al. (48)	2008	RS	11	100	ICA	BI	+		+	18
Nakagawa et al. (49)	2007	CR	1	100	ICA	BI			+	0
Edwards et al. (41)	2007	RS	110	100	ICA	BI	+		+	26
Archondakis et al. (50)	2007	RS	8	100	ICA	BI			+	0
Inaba et al. (51)	2006	PS	51	100	IJV, CCA	PI	+	+		0
Cohen et al. (52)	2005	RS	12	100	ICA	BI				0
Joo et al. (53)	2005	RS	10	100	ICA	BI			+	0
Cothren et al. (54)	2005	RS	46	100	ICA	BI	+		+	0
Fateri et al. (55)	2005	CR	1	100	ICA	BI			+	0
Self et al. (56)	2004	CR	1	100	ICA	PI			+	0
ul Haq et al. (57)	2004	CS	2	100	ICA/CCA	PI			+	0
Fusonie et al. (58)	2004	CR	1	100	ICA	BI			+	0
Layton et al. (59)	2004	CS	3	33	ICA	PI			+	0
Ahn et al. (60)	2004	CR	1	100	ICA	PI			+	0
Duncan and Fourie (61)	2003	CR	1	100	ICA	PI			+	0
Kubaska et al. (62)	2003	CS	3	66	ICA	BI/PI			+	0
Nanda et al. (63)	2003	RS	23	NR	CCA/ICA	BI/PI	+	+		26
Patel et al. (64)	2002	CR	1	100	ICA	PI			+	0
Duane et al. (65)	2002	CS	2	100	ICA	BI/PI			+	0
Navsaria et al. (66)	2002	RS	32	NR	CCA/ICA/ECA/IJV	PI		+		6.3
Brandt et al. (67)	2001	CS	6	100	ICA	BI			+	0
Scavee et al. (68)	2001	CR	1	100	ICA	BI			+	0
Redekop et al. (69)	2001	CS	6	66.6	ICA	BI/PI	+		+	0
Malek et al. (70)	2000	RS	10	20	ICA/CCA	BI			+	0
Kerby et al. (71)	2000	CR	1	100	ICA	BI			+	0
Coldwell et al. (72)	2000	RS	14	100	ICA	BI			+	0
Sekharan et al. (11)	2000	RS	145	100	NR	BI/PI	+	+		NR
Szopinski et al. (73)	2000	CR	1	100	ICA	PI			+	0
Liu et al. (74)	1999	RS	7	28.5	ICA	BI			+	0
Shames et al. (75)	1999	CR	1	100	ICA	BI			+	0
Parodi et al. (76)	1999	RS	29	27.5	CCA/ICA	BI/PI			+	0
Reiter et al. (77)	1998	CR	1	100	ICA	PI			+	0
Morotta et al. (78)	1998	CR	1	100	ICA	PI			+	0
Eachempati et al. (79)	1998	RS	23	100	ICA/CCA	BI			+	0
Klein et al. (80)	1997	CR	1	100	ICA	BI			+	0
Perez-Cruet et al. (81)	1997	CR	1	100	ICA	BI			+	0
Biffi et al. (82)	1997	RS + PS	312	38	NR	PI		+		35
Duke et al. (83)	1997	CS	6	100	ICA	BI			+	0
Bernstein et al. (84)	1997	CR	1	100	ICA	BI			+	0
Matsuura et al. (85)	1997	CR	1	100	ICA	BI			+	0
Colella et al. (86)	1996	RS	20	100	ICA	BI	+			0
Ramadan et al. (87)	1995	RS	82	66	CCA/ICA/VA	BI/PI	+	+	+	17
Schievink et al. (88)	1994	RS	22	9	ICA	BI		+		0

*NR, no reported; RS, retrospective study; CS, case series; CR, case report; ICA, internal carotid artery; CCA, common carotid artery; ECA, external carotid artery; IJV, internal jugular vein; VA, vertebral artery; BI, blunt injury; PI, penetrating injury*.

### Injury to the VA

An injury to the VA due to dissection, intimal disruption, pseudoaneurysm, or arteriovenous fistula from blunt trauma or active hemorrhage from penetrating wound could be safely evaluated with CTA in the hemodynamically stable patient (104). Blunt VA injuries were originally diagnosed by DSA using the same grading criteria (Table 1) that were applied to the VA as for CA. In contrast to blunt carotid injuries, the grade of blunt VA injuries does not relate to the rising risk of stroke. The stroke risk is standing at 20% irrespective of lesion grade (104).

Medical management has no role for PIs, but it does have a role for blunt injuries identified during the diagnostic evaluation. Symptomatic patients with posterior circulation stroke should be treated with heparin and monitored for hemorrhagic conversion with serial neurological examinations (104). In patients who could not tolerate anticoagulation, aspirin seems to be as effective as heparin. Miller et al. (37) presented their results of 43 VA injuries; 32 patients received aspirin or clopidogrel and 8 received only heparin. None of the patients developed stroke (37). The authors proposed that symptomatic patients and those without contraindications to anticoagulation should be treated with 3–6 months of anticoagulation. On the other hand, asymptomatic patients should be treated with either 3–6 months of anticoagulation or dual-antiplatelet therapy. However, data are still insufficient to safely recommend the abovementioned treatment.

The placement of endovascular stent for a pseudoaneurysm or intimal flap followed by coil embolization of an arteriovenous fistula has been described as an appropriate treatment in the literature (105). If the vessel is intact, the injury may be crossed from antergrade approach, allowing embolization of both the outflow and inflow. In nearly half of VA injuries evaluated with endovascular techniques, the vessel was thrombosed and required no treatment at all. Until today, there are no data supporting routine stenting for blunt VA injuries.

Open surgical approach is reserved for patients with active bleeding from VA at the time of neck exploration. Often, the control of VA bleeding can be challenging and, except ligation, postoperative endoluminal embolization is required (21). Unilateral surgical ligation results in a stroke rate of 3–5% (106). Deaths are invariably due to prehospital exsanguination or an associated injury to the brain.

### Injury to the IJV

Venous injuries to the IJV occur in 20% of the penetrating trauma. Isolated injuries are manifested with hard or soft signs of a vascular injury but patients are rarely unstable.

The surgical approach depends on the patient’s symptomatology. In case of hard signs, the IJV can be ligated. The patient should be monitored for cerebral edema but this is an uncommon event even with bilateral IJV ligation (107). As bilateral ligation of IJV may result in pseudotumor cerebri, every effort to maintain almost one IJV should be made (107). Simple laceration of the vein can be repaired with lateral venorrhaphy if less than 50% of the wall is involved. Extensive vein repairs using venous interposition or spiral panel grafts typically do not have a role in penetrating neck injuries when time is critical (108).

## Conclusion

Cervical vascular injury in zone II remains till today the most common following blunt/penetrating neck trauma posing the surgeon in the situation to make a rapid decision to salvage patient’s life. Hard signs and symptoms of vascular injuries after penetrating trauma require immediate surgical investigation while the traditional debate between obligatory investigation versus selective non-operative management with serial examination and further diagnostic testing seems gradually to be resolved in asymptomatic patients. Although the suitable treatment of BCAI is a matter of debate and strictly subjective, anticoagulation or antiplatelet therapy should be first administered in neurologically stable patients. If the neurological status of patients is worsening, despite adequate anticoagulation or if antithrombotic therapy is contraindicated, endovascular approach should be attempted in arterial lesions suitable for endovascular repair. Nonetheless, open repair is still indicated for the treatment of PIs and for patients unfit for endovascular intervention.

Despite important progress in the management of carotid trauma, prospective, randomized clinical studies are required to address important issues concerning the treatment of vascular injuries in zone II of the neck.

## Author Contributions

GK: project development, data collection, manuscript writing, and review manuscript. KM: data collection. CB: project development. SG: organized study and review the manuscript.

## Conflict of Interest Statement

The authors declare that the research was conducted in the absence of any commercial or financial relationships that could be construed as a potential conflict of interest. The reviewer GG declared a past co-authorship with the authors to the handling editor, who ensured that the process met the standards of a fair and objective review.
